# The Predictive Role of Low Spatial Frequencies in Automatic Face Processing: A Visual Mismatch Negativity Investigation

**DOI:** 10.3389/fnhum.2022.838454

**Published:** 2022-03-11

**Authors:** Adeline Lacroix, Sylvain Harquel, Martial Mermillod, Laurent Vercueil, David Alleysson, Frédéric Dutheil, Klara Kovarski, Marie Gomot

**Affiliations:** ^1^Univ. Grenoble Alpes, Univ. Savoie Mont Blanc, CNRS, LPNC, Grenoble, France; ^2^Defitech Chair in Clinical Neuroengineering, Center for Neuroprosthetics and Brain Mind Institute, EPFL, Geneva, Switzerland; ^3^Grenoble Institut Neurosciences, InsermU1216, CHU Grenoble, Grenoble, France; ^4^Université Clermont Auvergne, CNRS, LaPSCo, CHU Clermont-Ferrand, Clermont-Ferrand, France; ^5^Hôpital Fondation Rothschild, I3N, Paris, France; ^6^Université de Paris, INCC UMR 8002, CNRS, Paris, France; ^7^UMR 1253 iBrain, Université de Tours, Inserm, Tours, France

**Keywords:** vMMN, spatial frequencies, face processing, predictive coding, prediction error, automatic visual processing

## Abstract

Visual processing is thought to function in a coarse-to-fine manner. Low spatial frequencies (LSF), conveying coarse information, would be processed early to generate predictions. These LSF-based predictions would facilitate the further integration of high spatial frequencies (HSF), conveying fine details. The predictive role of LSF might be crucial in automatic face processing, where high performance could be explained by an accurate selection of clues in early processing. In the present study, we used a visual Mismatch Negativity (vMMN) paradigm by presenting an unfiltered face as standard stimulus, and the same face filtered in LSF or HSF as deviant, to investigate the predictive role of LSF vs. HSF during automatic face processing. If LSF are critical for predictions, we hypothesize that LSF deviants would elicit less prediction error (i.e., reduced mismatch responses) than HSF deviants. Results show that both LSF and HSF deviants elicited a mismatch response compared with their equivalent in an equiprobable sequence. However, in line with our hypothesis, LSF deviants evoke significantly reduced mismatch responses compared to HSF deviants, particularly at later stages. The difference in mismatch between HSF and LSF conditions involves posterior areas and right fusiform gyrus. Overall, our findings suggest a predictive role of LSF during automatic face processing and a critical involvement of HSF in the fusiform during the conscious detection of changes in faces.

## 1. Introduction

### 1.1. Spatial Frequency in Face Processing

Visual processing of faces is a complex mechanism, relying on high and low level cognitive functions. Among the later, from the first steps of visual perception of faces, visual recognition involves spatial frequencies (SF) processing (e.g., Vuilleumier et al., 2003; Goffaux and Rossion, 2006; Goffaux et al., 2011). SF refer to a spectrum of spatial information in an image, expressed as a number of cycles per degree (cpd) of visual angle and derived from the Fourier transform (Morrison and Schyns, 2001; Park et al., 2012; Bachmann, 2016). While low spatial frequencies (LSF) convey coarse information mainly through the dorsal stream, high spatial frequencies (HSF) convey fine details through the ventral stream (see Skottun, 2015).

How are LSF and HSF involved in processing faces? This question has been extensively studied, but results are mixed and not conclusive (for reviews see Ruiz-Soler and Beltran, 2006; Jeantet et al., 2018). Indeed, SF preference depends on the task (Schyns and Oliva, 1999; Ruiz-Soler and Beltran, 2006; Smith and Merlusca, 2014), at least in behavioral studies. For example, emotion detection and gender categorization would not be carried by the same SF (Schyns and Oliva, 1999). Emotion categorization would rely more on LSF, particularly at the early stages (e.g., Schyns and Oliva, 1999; Mermillod et al., 2005, 2010; Wang et al., 2021; but Deruelle et al., 2004; Jennings et al., 2017). Nevertheless, this pattern can be reversed with additional task constraints, such as an interference effect (Lacroix et al., 2021; Shankland et al., 2021; but Beffara et al., 2015) or the complexity of the emotion (Cassidy et al., 2021), which leads to rely more on HSF. The type of emotional content (Kumar and Srinivasan, 2011; Wang et al., 2015) as well as the awareness of the stimulus (De Gardelle and Kouider, 2010), but also individuals differences (Dube et al., 2014; Langner et al., 2015), would also influence the preference in SF processing. However, there is a large body of evidence using neuroimagery indicating that faces are usually processed in a coarse-to-fine manner, with LSF being processed faster than HSF (e.g., Halit et al., 2006; Hegdé, 2008; Nakashima et al., 2008; Vlamings et al., 2009; Goffaux et al., 2011; Tian et al., 2018; Petras et al., 2019, 2021). The efficiency of coarse-to-fine processing has also been demonstrated in computer vision (e.g., Zhou et al., 2013; Zhang et al., 2014).

Within a predictive coding framework (Rao and Ballard, 1999), Bar et al. (2006) suggested a neurocognitive model of the coarse-to-fine processing. LSF would be quickly extracted and transmitted to the orbitofrontal cortex where predictions would be formed (Bar et al., 2006; Bar, 2007). These predictions would then be sent back to infero-temporal areas (Kveraga et al., 2007), guiding the processing of details extracted from HSF information by a top-down process and facilitating fast recognition (Bar et al., 2006; Bar, 2007). This predictive brain model of visual perception is supported by a study in magnetoencephalography showing activation of the orbitofrontal cortex beginning at 80 ms and synchronizing with the fusiform gyrus around 130 ms, driven by LSF, during object recognition (Bar et al., 2006). Regarding face processing, recent findings showing that informative LSF modulate the processing of HSF during passive viewing of faces (Petras et al., 2019, 2021), were also in accordance with this model. However, to our knowledge, there is no neuroimaging study investigating more specifically Bar's model, that is, the predictive role of LSF, during face processing. As visual stimuli such as faces are processed automatically, at the pre-attentive level (Palermo and Rhodes, 2010; Kovarski et al., 2019), the task would not implicate any instruction nor explicit recognition.

### 1.2. Visual Mismatch Negativity

Pre-attentive visual processing can be investigated with oddball paradigms, during which rare deviant stimuli are presented within a stream of frequent standard stimuli. With this type of paradigm, automatic change detection is measured with the Mismatch Negativity (MMN), a component that has been initially recorded in the auditory modality (Näätänen et al., 1978) but can also be elicited within the visual and somatosensory modalities. The visual MMN (vMMN) is a differential negative event-related potential (ERP) representing the pre-attentive neural mechanism involved in the automatic detection of unpredicted visual changes among a learned regularity (Czigler et al., 2002, 2006; Stefanics et al., 2015). In line with predictive coding framework (Rao and Ballard, 1999; Friston, 2005, 2010), vMMN is often considered as a neural correlate of prediction error (Friston, 2005; Garrido et al., 2009; Stefanics et al., 2014, but May, 2021; O'Reilly and O'Reilly, 2021) i.e., the difference between sensory input and predictions, based on our internal model constructed upon the regularity of standard stimuli. Thus, it appears to be particularly suitable to investigate predictive processes. vMMN is usually observed in a wide time-window between 100 and 500 ms depending on the studies, and it includes one (e.g., Tales et al., 1999) or two deflections (e.g., Heslenfeld, 2003; Czigler et al., 2006). While posterior activity is systematically observed (Kimura et al., 2010; Urakawa et al., 2010; Cléry et al., 2013), vMMN can also be found later in temporal regions (Heslenfeld, 2003; Kuldkepp et al., 2013). Additionally, a central positivity is sometimes observed (e.g., Czigler et al., 2006; Cleary et al., 2013; File et al., 2017). An fMRI study investigating the brain correlates of automatic visual change detection to shapes, found greater brain activation in response to deviant stimuli compared to standard stimuli in a wide network including the left posterior parietal, anterior pre-motor and superior occipital cortices, the left medial frontal, as well as the orbitofrontal gyri and the visual dorsal and ventral streams (Cléry et al., 2013). These results show involvement of both areas dedicated to visual perception and areas related to pre-attentional processing of change detection.

vMMN has been observed in a broad range of tasks, at different levels of visual processing. Thus, vMMN is elicited during change detection of color (Liu and Shi, 2008; Urakawa et al., 2010), line orientation (Yan et al., 2017), shape (Cléry et al., 2013), motion (Kuldkepp et al., 2013; Schmitt et al., 2018; Rowe et al., 2020), and spatial frequencies (Heslenfeld, 2003; Sulykos and Czigler, 2011; Cleary et al., 2013; Susac et al., 2014). However, so far, vMMN studies on spatial frequencies changes did not really investigate the contrast in response to deviant stimuli in HSF vs. in LSF. For instance, in Cleary et al. (2013), standards were always HSF gratings and deviants LSF gratings. Sulykos and Czigler (2011) used gratings but did not compare the response to HSF vs. LSF gratings as the authors were interested in the additive effect of two deviant features (orientation and spatial frequencies) and in the visual field. Heslenfeld (2003), however, compared deviance response to HSF vs. LSF gratings but did not find any interaction between deviance and spatial frequencies, whereas Susac et al. (2014) found opposite polarities for vMMN response to HSF compared to LSF, with opposite orientation of sources as well.

vMMN has also been observed for socially relevant changes such as facial emotion (Astikainen and Hietanen, 2009; Astikainen et al., 2013; Kreegipuu et al., 2013; Kovarski et al., 2017; Chen et al., 2020), gender (Kecskés-Kovács et al., 2013), attractiveness (Zhang et al., 2018), or identity (Rossion et al., 2020). However, vMMN in response to different spatial frequencies has never been studied with complex stimuli, such as scene, objects, or faces. Yet, investigating vMMN elicited by spatially filtered faces could help to further investigate which spatial frequency band is mainly involved in processing faces at a pre-attentive level, and more specifically the predictive role of LSF information.

### 1.3. Aim and Hypotheses

The aim of the current study was to determine to what extend LSF or HSF generate predictions in an intrinsically predictive task (i.e., an oddball task) involving automatic face processing. To do so, participants had to perform a concurrent task maintaining their attention toward the stimuli but allowing their implicit processing (Flynn et al., 2016; Kovarski et al., 2017; Male et al., 2020). This task should not favor global or local perception so that it would not influence the processing toward HSF or LSF. Here, we designed an oddball task involving a gray-scale unfiltered neutral face as a standard stimulus, the same face in color as target (as it involves both ventral and dorsal streams and thus will not orient the processing toward LSF or HSF; Claeys et al., 2004) and the same gray-scale face filtered in HSF or LSF as deviant stimuli.

Additionally, we used an equiprobable sequence as a control condition to deal with adaptation/refractoriness and differences in physical features (Grill-Spector et al., 2006; Li et al., 2012; Stefanics et al., 2014; Kovarski et al., 2017; Male et al., 2020). Indeed, contrary to typical vMMN paradigm which usually compare physically different stimuli (deviant vs. standard) leading to the impossibility to disentangle response to regularity violation from response related to the physical differences between stimuli, this control paradigm enables the comparison of identical stimuli (Garrido et al., 2009; Stefanics et al., 2014; Fitzgerald and Todd, 2020).

We capitalized on the fact that less predictable (more surprising deviants) would elicit more negative amplitude (see Stefanics et al., 2014), in accordance with the notion of prediction error signaling. Based on this account and on Bar's model (Bar et al., 2006), we hypothesized that HSF would elicit larger vMMN response than LSF as the latter are supposed to be at the root of predictive process in visual perception. In other words, prediction from LSF in the unfiltered stimulus would match the LSF deviant, but not the HSF deviant, eliciting a larger prediction error in the later case.

## 2. Materials and Methods

### 2.1. Participants

Thirty-four healthy adults (18 females; Mean age ± SD [range] = 29.4 ± 7.5 [19.5–46.0]) with no psychiatric or neurological disorder, participated in this study. Visual acuity was tested using the Landolt C task of the Freiburg Vision Test (FrACT3), version 3.10.5 (Bach, 1996). All participants had a logMAR <0.10. Participants gave their written informed consent after being provided with information on the study's objectives and procedures. The study was approved by the Ethics Committee (Comite de Protection des Personnes Ile de France 1—IRB/IORG: IORG0009918) under agreement number 2019-A01145-52. Participants received monetary compensation for their participation. EEG acquisitions were performed at IRMaGe neurophysiology facility (Grenoble, France).

### 2.2. Stimuli and Procedure

**Figure 1:**
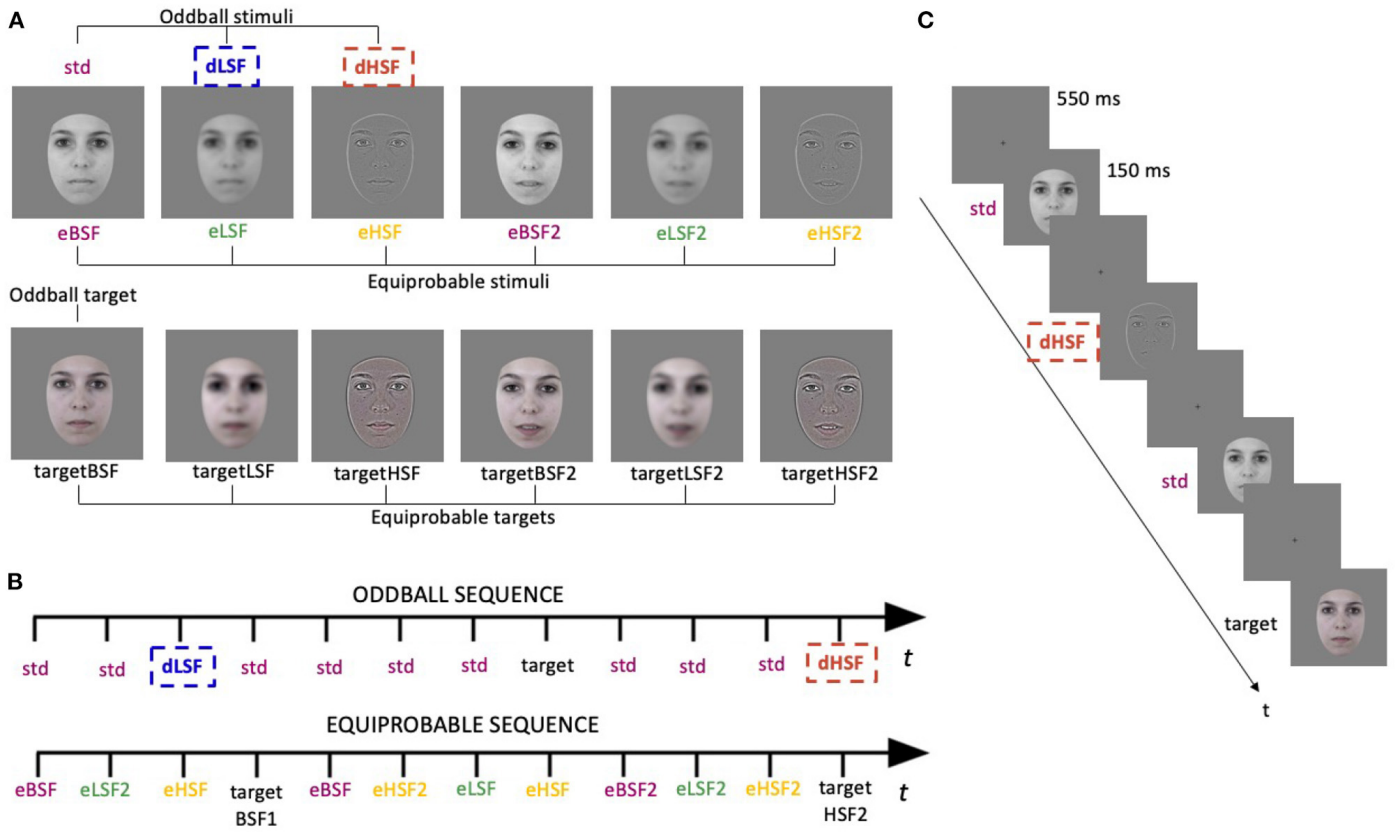
Stimuli and procedure. **(A)** The first line represent the gray-scale stimuli used in the oddball sequence (std, standard; dLSF, deviant Low Spatial Frequency; dHSF, deviant High Spatial Frequency) and in the equiprobable sequence (eBSF, equiprobable Broad Spatial Frequency; eLSF, equiprobable Low Spatial Frequency; eHSF, equiprobable High Spatial Frequency). The second line represents the colored target stimuli for the oddball sequence (first face) and for the equiprobable sequence (all faces). **(B)** Illustration of oddball and equiprobable sequences. **(C)** Task schematic of the oddball sequence.

The procedure and stimuli were previously used and behaviorally validated (see Kovarski et al., 2017), but emotional deviants were replaced by spatially filtered deviants. Stimuli were photographs of two neutral faces of the same actress (Figure 1A) presented in an oddball and an equiprobable sequences (Figure 1B). In the oddball sequence, the standard stimulus was a grey-scale unfiltered face presented with a probability of occurrence of *p* = 0.80. The deviant stimuli were the same photograph either filtered in LSF (dLSF; *p* = 0.10) or filtered in HSF (dHSF; *p* = 0.10). LSF images contained only SF below 1.5 cycles per degree (cpd; 8.7 cycles per faces) and HSF images contained only SF above 6 cpd (34.2 cycles per faces). These cutoffs were chosen as SF preferentially used in face processing range from 4.5 to 37 cycles per faces (for a review see Jeantet et al., 2018) and authors who investigated SF in face processing previously used similar cutoffs (e.g., Goffaux and Rossion, 2006; Goffaux et al., 2011; Beffara et al., 2015). Filtered images were obtained by fast Fourier transform and by multiplying the Fourier energy with Gaussian filters. Images were normalized to obtain a mean luminance of 0.5 (for luminance values between 0 and 1) with a standard deviation of 0.075 (root mean square contrast). SF filtering and normalization were elaborated using MATLAB (Mathworks Inc., Sherborn, MA, USA). The target stimuli (*p* = 0.05 among standard stimuli) were also the same photograph but colored, so that it did not favor HSF or LSF processing. Saturation of the colored image was lowered to reduce the salience among stimuli and maintain attention. Then, color images were filtered based on luminance (*L*)-chrominance(*Chr*) decomposition (*L* = (*R*+*G*+*B*)/3 and chrominance *Chr* = [*R*−*L, G*−*L, B*−*L*]). Only the luminance *L* was filtered either low-pass or high-pass and the chrominance was added back to the filtered luminance with a multiplication factor of 3/5 to decrease its variance.

The oddball sequence (Figure 1B) comprised 1,575 stimuli, presented in two sessions of 10 min each. In the equiprobable sequence (Figure 1B), the three stimuli of the oddball sequence (renamed eBSF—for Broad Spatial Frequencies/unfiltered stimuli—, eHSF and eLSF) as well as three additional stimuli (eBSF2, eLSF2, and eHSF2) of another neutral expression of the same actress (with the mouth slightly opened—Figure 1A) were presented pseudo-randomly, avoiding immediate repetition, at a probability of occurrence of ~0.16. In this sequence, target stimuli were the same stimuli but colored (*p* = 0.05; *p* ≈ 0.01 each). This sequence comprised 835 stimuli presented in one session of 10 min. Oddball and equiprobable sequences order was counterbalanced, as well as the two oddball sequences.

While participants sat comfortably in an armchair, stimuli were displayed centrally on a CRT screen (37 × 29.6 cm; refresh rate = 75 Hz; resolution = 1,280 × 1,024 pixels) at a viewing distance of 87 cm so that the faces corresponded to 5.8° of visual angle. Stimuli were presented using Presentation® software (Neurobehavioral Systems, Inc., Berkeley, CA, www.neurobs.com) for 150 ms with a 550 ms inter-stimulus interval (Figure 1C). Participants were instructed to look at the fixation cross and to press a button as quickly as possible when they saw a colored face. All subjects were monitored with a camera during the recording session.

### 2.3. Behavioral Data Collection and Analysis

Hit rate, false alarm, miss, and correct rejection of the target detection were recorded during the experiment. The sensitivity index *d*′= (z-score hit rate) − (z-score false alarm rate) was calculated with the psycho package (Makowski, 2018) on R version 4.0.3 (R Core Team, 2020) and R studio version 1.3.1075 (RStudio Team, 2019) to evaluate the involvement of the participants in the task.

### 2.4. EEG Data Collection and Analysis

#### 2.4.1. EEG Recording

EEG data were recorded using a 96 active electrodes system (BrainAmp amplifiers and EasyCaps, Brain Products GmbH, Germany) following the 10-5 standard system. Electrooculographic (EOG) activity was recorded using two electrodes on the left and right outer canthi of the eyes and two above and below the left eye for spotting horizontal and vertical eye movements respectively (hEOG and vEOG). The ground electrode for the EOG was placed on the left base of the neck. Impedance were adjusted and kept below 25 kΩ before and during the recording. Signal was recorded with a sampling rate of 1,000 Hz, using an anti-aliasing filter at 500 Hz. FPz and FCz were defined as the ground and reference electrodes, respectively.

#### 2.4.2. EEG Pre-processing

EEG pre-processing and analysis were performed using Brainstorm software (Tadel et al., 2011), and other custom scripts developed in MATLAB (The MathWorks Inc.). First, bad channels were visually inspected and removed during the recording and during the pre-processing for each participant, based on both temporal (deviants dynamics, flat signals) and frequency (deviant Welch's power spectrum density) characteristics. Time periods contaminated by high-frequency muscular artifacts were discarded manually. We then re-referenced the signal using average reference. Both horizontal and vertical eyes movements artifacts were targeted by analyzing the corresponding EOG recording, and corrected by applying a specific signal-space projection (SSP, a spatial decomposition method to be compared with independent component analysis, Uusitalo and Ilmoniemi, 1997). To do so, hEOG and vEOG signals were band-pass filtered between 1.5 and 20 Hz or 40 Hz, respectively, and then normalized using z-score. Any time period containing data above two standard deviations was considered as an artifact of ocular movements. SSP was then computed on the −200 to 200 ms time window relative to the artifact onset. The resulting SSP component relative to eyes movements was finally detected and rejected from the signal. The clean signal was band-pass filtered using cutoffs of 0.1 and 40 Hz. Time series of the rejected channels were interpolated using their neighboring channels. Finally, trials were epoched over a 700 ms analysis period, from 100 ms pre-stimulus to 600 ms post-stimulus. After pre-processing, a total of 0.2 % of the trials were discarded.

#### 2.4.3. Event-Related Potentials

The first three trials of the sequence as well as trials occurring after target or deviant stimuli were excluded from ERP processing and analyses. Each ERP was computed by averaging all the trials of each stimulus of interest from the oddball sequence (standard, dHSF, dLSF) and from the equiprobable sequence (eBSF, eHSF, eLSF) and standardized using z-score against baseline (taken prior to stimulus onset from −100 to −1 ms) for each subject. Non-standardized and standardized ERP of each participant on each condition of interest were visually inspected. Again, any remaining deviant electrode was discarded and interpolated using its neighboring channels. In the end, a mean of five channels (Range = 0–18) on 96 were interpolated by participant. vMMNs for HSF and LSF were calculated as the arithmetic difference between ERPs to deviant and to equiprobable stimuli, taken from oddball and equiprobable sequences (dHSF-eHSF and dLSF-eLSF, respectively). Grand average difference waveforms were finally computed across participants.

#### 2.4.4. Source Level Analysis

Source reconstruction was performed to estimate the anatomical location of electric sources that could explain the activities recorded on the scalp. It was performed with the sLoreta method (standardized low-resolution brain electromagnetic tomography) on the ICBM152 brain template using a volumic head model. The model was computed employing the symmetric boundary element method elaborated in the openMEEG freeware, using the default values for conductivity and layer thickness (Gramfort et al., 2010). For each participant, we calculated the noise covariance matrices from the concatenation of all the baseline periods (i.e., −100 to −1 ms before the onset of stimuli). Source activities were reconstructed on each of the 15,000 cortical vertices using sLoreta. Individual source maps were normalized against baseline (z-score) and averaged across subjects to obtain final group maps. They were used to show the potential sources of significant clusters, by averaging activities in the corresponding time windows.

### 2.5. Statistics

#### 2.5.1. ERPs Analyses

**Figure 2:**
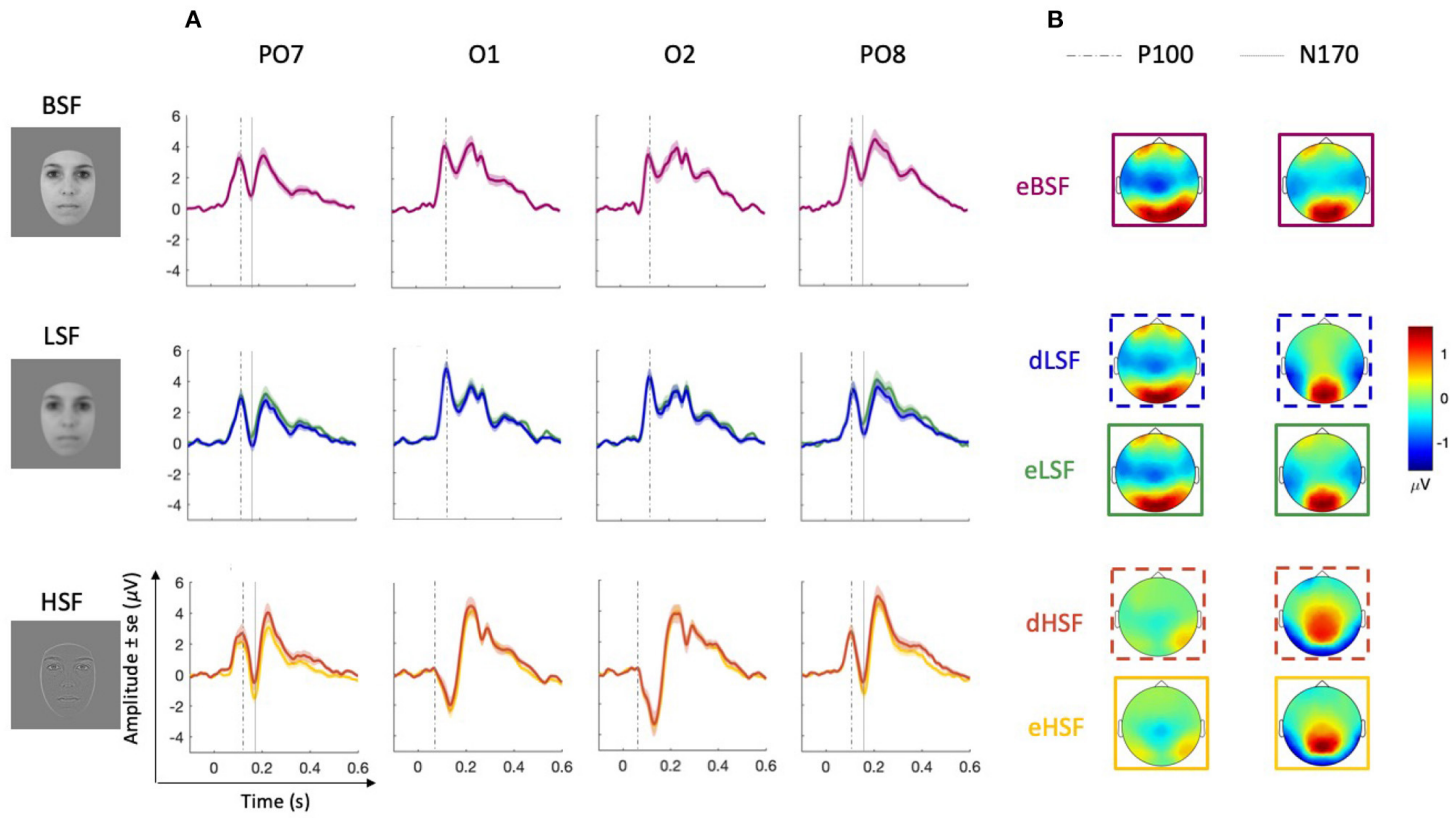
Sensory responses. **(A)** Grand average ERPs for each equiprobable (eBSF in purple, eLSF in green and eHSF in yellow) and deviant (dLSF in blue and dHSF in orange) condition over selected occipital (O1 and O2) and parieto-occipital (PO7 and PO8) electrodes. Dotted lines on O1 and O2 represent the latencies of P100 scalp topographies; dotted lines on PO7 and PO8 represent the latencies of N170 topographies (for the latest line) and of the latencies of the P100 used in statistical analyses (for the earliest line). **(B)** Scalp topographies showing activity at the peak used for P100 (on O1 and O2) and N170 (on PO7 and PO8).

In order to assess the sensory response to filtered and unfiltered equiprobable stimuli (eHSF, eLSF, and eBSF), we investigated ERP components. We extracted peak amplitude for each participants using MATLAB scripts (based on *findpeaks* function) and visual inspection over the latency range of 60–140 ms on O1 and O2 and on PO7 and PO8 for the P100. However, as a negative peak was observed for P100 in the HSF condition (Figure 2A), we performed P100 analysis on PO7 and PO8 only. For the N170, P100–N170 peak-to-peak difference was performed by measuring peaks in the latency ranges of 60–140 and 130–200 ms on PO7 and PO8. Then, data were analyzed with repeated measure analyses of variance (ANOVA) on RStudio Team (2019) using the afex package (Singmann et al., 2021) with Huynh-Feldt correction in case of departure of sphericity (tested with Mauchly tests). Analyses included SF (eBSF, eLSF, eHSF) and channels/hemisphere (PO7 vs. PO8) as within subject factors. *Post-hoc* tests were performed with the emmeans package (Lenth, 2021) by applying a Bonferroni correction. However, in case of strong violation of assumptions (normality and sphericity; which was the case for most analyses), we ran non-parametric tests, i.e., Friedman rank sum test for SF (with Durbin-Conover test for pairwise comparison, Holm corrected) and Wilcoxon signed-rank test for channels.

#### 2.5.2. Cluster Based Statistics

To investigate vMMN, cluster based permutation tests (using ft_timelock statistic, with “Monte-Carlo” and cluster as parameters) were used to assess differences between conditions (dHSF vs. eHSF, dLSF vs. eLSF, and dHSF-eHSF vs. dLSF-eLSF) regarding scalp EEG data. Samples were selected for clustering with a significance threshold α = 0.05 using dependent paired two-tailed *t* test over the 0–600 ms time window after stimulus onset on all electrodes. Significant samples were included in the clustering algorithm with the requirement of a minimum of two neighboring channels. Then, cluster-level statistics were calculated by summing the *t* values within each cluster and Monte-Carlo procedure (1,000 permutations) was used for correction. The significance threshold for clusters was set to *p*_*cluster*_ < 0.05.

## 3. Results

### 3.1. Behavioral Results

*D'* values indicated a good compliance to the task (Mean *d'* = 4.52 ± 0.83). Nevertheless, three participants had a high miss rate (between 25 and 40%). Their recordings were visually inspected to ensure that they processed the visual stimuli. P100 were present in their recordings suggesting basic face processing and compliance to the task. Consequently, they were included in the analyses.

### 3.2. Event Related Potentials

#### 3.2.1. P100

Figure 2A shows grand average ERPs (in μV) at O1 and O2 in each condition whereas Table 1 shows mean amplitude (in z-score) and latencies. While there is a large positive peak in LSF and BSF around 117 ms (P100), this is not observed in the HSF condition on occipital electrodes but on parieto-occipital channels. Instead, a negative peak is observed in the HSF condition occurring at 131 ms. Figure 2B shows differences in topographies in HSF compared to LSF and BSF that could explain the difference in ERPs. Whereas, there is large positivity over the occipital areas in LSF and BSF, it appears reduced in HSF, and does not involve the most posterior areas. We also observe frontal activation in LSF and BSF which is not observed in HSF.

**Table 1:** Mean amplitude (in z-score) and latencies (in milliseconds), with standard deviation (sd) underneath, for P100 and N170.

	**P100**								**N170**			
	**Amplitudes (σ)** **±sd**				**Latencies (ms)** **±sd**				**Amplitudes (σ)** **±sd**		**Latencies (ms)** **±sd**	
	**O1**	**O2**	**PO7**	**PO8**	**O1**	**O2**	**PO7**	**PO8**	**PO7**	**PO8**	**PO7**	**PO8**
BSF	12.31	12.88	12.60	12.59	118	117	113	108	0.25	2.39	166	159
	7.85	8.34	9.21	7.83	13	17	20	19	6.50	7.84	14	18
LSF	17.57	14.03	12.01	11.41	118	113	113	109	0.40	0.87	165	165
	10.86	10.70	10.75	7.75	15	16	20	20	6.90	6.64	15	18
HSF	/	/	11.69	10.14	/	/	104	101	−5.46	−7.21	163	157
	/	/	8.46	7.12	/	/	22	16	10.38	14.46	19	18
SF effect	/		ns		/		*p* = 0.008		*p* = 0.001		ns	
Channel effect	/		ns		/		ns		ns		*p* = 0.025	

*The last line represents the p-value associated with the main effect of spatial frequencies (simple effects are described in section 3) and with the main effect of channel. BSF, broad spatial frequencies; LSF, low spatial frequencies; HSF, high spatial frequencies; SF, spatial frequencies; ns, non significant*.

Analysis of P100 amplitudes on PO7 and PO8 revealed no significant effect of SF nor hemisphere.

Analyses of P100 latencies on PO7 and PO8 revealed a significant effect of SF on P100 latencies [$\chi_{F}^{2}\left(2\right)=9.59$, *p* = 0.008, *W* = 0.14], P100 latencies being shorter for eHSF than for eLSF (*p* = 0.006). The effect of hemisphere (O1 and O2) on P100 latency was not significant.

#### 3.2.2. N170

Topographies of N170 shows parieto-occipital activity (in μV) in the three SF conditions (Figure 2B). Mean amplitudes (in z-score) as well as latencies are reported in Table 1. Visual inspection (on Figure 2A) of ERP on PO7 and PO8 confirms a large decrease of the amplitude following the P100, around 170 ms, corresponding to the N170, and which appears as more negative in the HSF condition.

Analyses on N170 peak-to-peak amplitudes (on PO7 and PO8) revealed an effect of SF [$\chi_{F}^{2}\left(2\right)=13.94$, *p* < 0.001, *W* = 0.21]. eHSF elicited a larger N170 than eBSF (*p* < 0.001) and than eLSF (*p* = 0.013), but there was no significant difference between eBSF and eLSF. The effect of hemisphere on N170 amplitude was not significant. Additionally, whereas there was no effect of SF on N170 latencies, the effect of hemisphere was significant [*F*_(1, 33)_ = 5.51, *p* = 0.025, $\eta_{p}^{2}=0.14$] with the N170 appearing earlier on the right (PO8) than on the left hemisphere (PO7).

### 3.3. Cluster Based Statistics

#### 3.3.1. Visual Inspection of Mismatch Response

**Figure 3:**
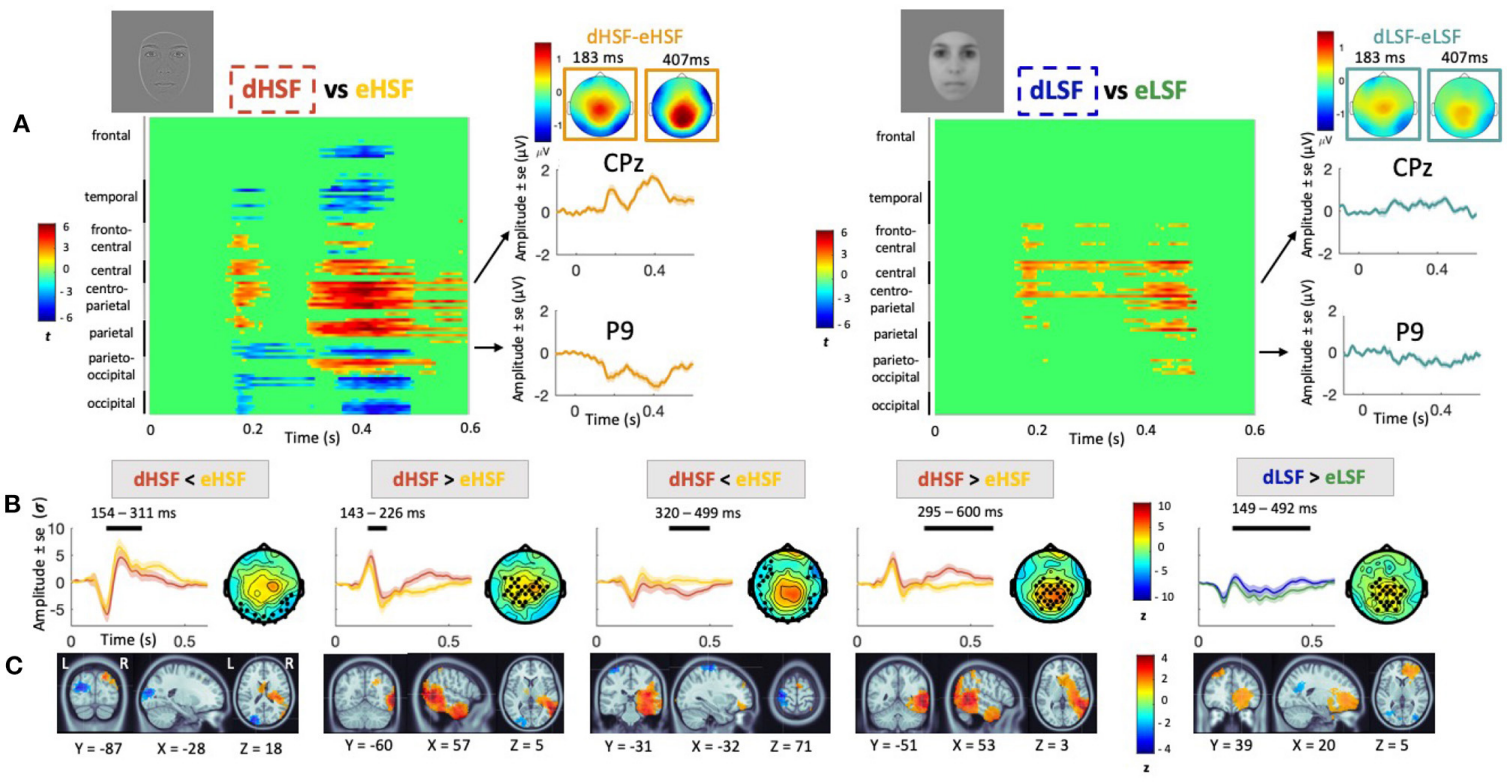
Visual mismatch response in HSF and LSF conditions. **(A)** Cluster analyses showing statistical significance for each condition (HSF mismatch response on the left and LSF mismatch response on the right) over the entire scalp in the 0–600 ms latency range; grand average mismatch response (in μV) at CPz and P9 channels over 0–600 ms latency range (in orange for HSF and in blue for LSF) with scalp topographies at the two peaks (183 and 407 ms). **(B)** Average waveforms (in σ) over the significant cluster's channels in the 0–600 ms latency range. Significant temporal window are represented with a black line over each waveform. For HSF, deviant condition is in orange and equiprobable in yellow. For LSF condition, deviant is in blue and equiprobable in green. Scalp topographies at the peak activity of the cluster are represented beside the waveforms. Black dots indicate electrodes belonging to significant clusters. **(C)** Source activity (in σ) averaged over the corresponding cluster's time window with MNI coordinates.

Figure 3 represents the visual mismatch response in HSF and LSF conditions. Visual inspection of grand average mismatch response at centro-parietal (CPz) and lateral (P9) sites allows to identify two peaks around 183 and 407 ms, respectively, especially in the HSF condition. The peaks are negative over parietal areas, corresponding to the vMMN, and positive over centro-parietal areas. In the LSF condition, the mismatch response appears smaller and more sustained (i.e., the peaks are less identifiable). Scalp topographies of the mismatch response at the two peaks latencies show occipital and parieto-ocipital negativity (vMMN) as well as centro-parietal positivity, both more pronounced in the HSF than in the LSF condition.

#### 3.3.2. HSF Mismatch Response

Visual observations were confirmed by statistical analyses showing two significant positive peaks over centro-parietal areas and two significant negative peaks over occipital areas in the HSF condition (Figure 3). More precisely, analysis revealed a first significant increased amplitude in dHSF relative to eHSF over centro-parietal areas from 143 to 226 ms (*p*_*cluster*1_ = 0.03). Source reconstruction indicated that the difference in activity was generated in the right fusiform area (BA37). There was a second significant increased amplitude in dHSF relative to eHSF over centro-parietal areas from 295 to 600 ms (*p*_*cluster*2_ = 0.002). Source reconstruction indicated that the difference in activity was related to a network including the right fusiform (BA37), the right anterior cingulate cortex (BA24) and the orbitofrontal cortex (BA11), passing by the right insula (BA13).

Additionally, we observed a significant decreased amplitude in dHSF relative to eHSF over occipital areas from 154 to 311 ms (*p*_*cluster*1_ = 0.02). Source reconstruction associated to this difference indicated generators in the left occipital areas (BA19; in the extrastriate cortex). A second decreased amplitude in dHSF relative to eHSF was observed over occipital and fronto-parietal areas from 320 to 499 ms (*p*_*cluster*2_ = 0.002) with generators of the mismatch located in the primary somotaosensory cortex (BA1).

#### 3.3.3. LSF Mismatch Response

Visual observation of a more sustained activity in LSF was also confirmed by statistical analysis. Indeed, we found a significant increased amplitude in dLSF relative to eLSF over centro-parietal areas from 149 to 492 ms (*p*_*cluster*_ = 0.002). The source of the mismatch was generated in the right anterior prefrontal cortex (BA10), but no activation was found in the fusiform for the LSF mismatch condition. Additionally, we did not find any cluster where dLSF amplitude was significantly inferior to eLSF.

#### 3.3.4. Contrast Between HSF and LSF Conditions

**Figure 4:**
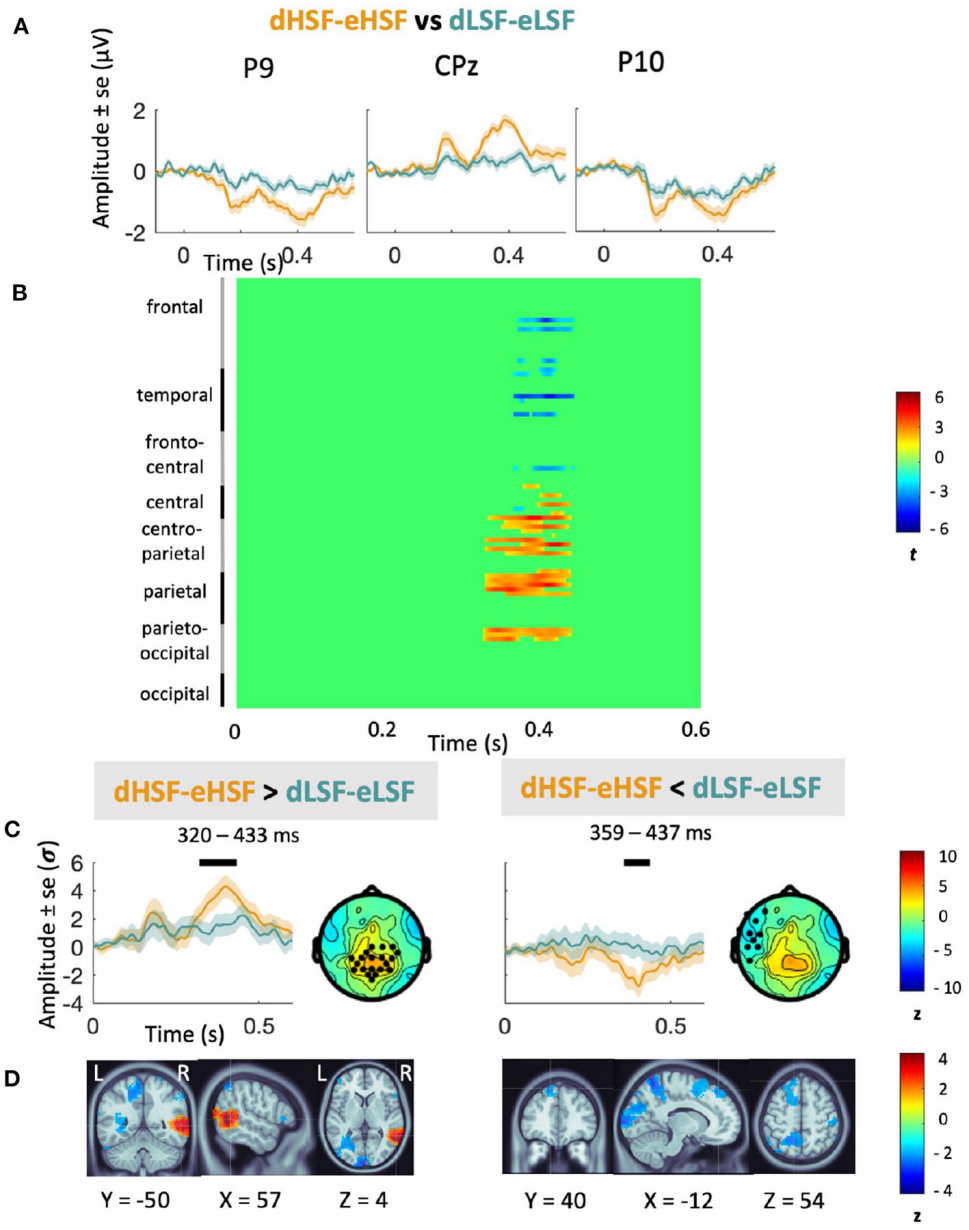
Contrast between HSF and LSF mismatch responses. **(A)** Grand average mismatch response (in μV) at P9, P10, and Cpz elicited by HSF (orange) and LSF (blue) deviants compared to their equivalent in the equiprobable condition and scalp topographies at the two peaks of activity. **(B)** Cluster analyses showing statistical significance for the contrast between HSF vMMN (in orange) and LSF vMMN (in blue) over the entire scalp in the 0–600 ms latency range. **(C)** Average waveforms (in σ) over the significant cluster's channels in the 0–600 ms latency range. Significant temporal window are represented with a black line over each waveform. Scalp topographies at the peak activity of the cluster are represented beside the waveforms. Black dots indicate electrodes belonging to significant clusters. **(D)** Source reconstruction for the significant clusters with activity averaged over the corresponding time window with MNI coordinates.

Analyses on the contrast between the mismatch responses (represented on Figure 4) revealed that amplitude of dHSF-eHSF was significantly superior to amplitude of dLSF-eLSF over centro-parietal areas from 320 to 433 ms (*p*_*cluster*_ = 0.004), i.e., for the second peak only. Source reconstruction indicated that this difference was associated with a larger positive activity in the right fusiform (BA37). Additionally, amplitude of dHSF-eHSF was inferior to dLSF-eLSF over left fronto-parietal areas from 359 to 437 ms (*p*_*cluster*_ = 0.02), i.e., again, for the second peak only. Source reconstruction indicated that this difference was associated with a larger negative activity in the left middle occipital gyrus (BA39), in the visual association area (BA18) and in the frontal cortex (BA8).

## 4. Discussion

In the present study, we investigated the involvement of LSF and HSF in predictive processes during automatic face processing. We used a controlled vMMN paradigm with unfiltered faces as standard stimuli and LSF and HSF filtered faces as deviants. The results showed that the vMMN was larger for HSF faces than for LSF faces, revealing lower prediction error for LSF than for HSF. These results suggest a critical role of LSF in visual prediction during automatic face processing, in accordance with Bar's model (Bar et al., 2006). Our investigation of sensory response at the early stages of face processing is also in line with a coarse-to-fine processing and provides additional evidence on this subject (e.g., Halit et al., 2006; Hegdé, 2008; Vlamings et al., 2009; Goffaux et al., 2011; De Moraes et al., 2016; Petras et al., 2019, 2021).

### 4.1. The Predictive Role of LSF Supported by vMMN

Visual inspection of mismatch ERPs on P9 and CPz as well as topographies (Figure 3) revealed a biphasic response over the occipital and parieto-occipital areas, but also over centro-parietal areas. This was particularly marked in the HSF condition. The biphasic response in the LSF condition was less clear, although the difference in activity appeared more sustained as confirmed by cluster analyses.

In the HSF condition, a significantly more negative amplitude for deviant compared to equiprobable was found in two time windows over posterior areas. This corresponds to the vMMN and would reflect the prediction error elicited by HSF deviants when occurring in a stream of expected standard stimuli. This biphasic response is consistent with previous studies investigating face-related vMMN (Astikainen and Hietanen, 2009; Kimura et al., 2012; Li et al., 2012; Kovarski et al., 2017) but other studies found a more sustained activity, i.e., with less identifiable peaks (Kecskés-Kovács et al., 2013; Kreegipuu et al., 2013). The difference in activity might be related to the stimuli. Kovarski et al. (2017) showed that the two steps vMMN are elicited by neutral and emotional stimuli, but that only emotional stimuli implicated a sustained activity. More generally, the experiments by File et al. (2017) suggested that the pattern of vMMN response varies according to the type of stimulus and level of deviance. Source reconstruction revealed that the vMMN to HSF was associated with activity in the extrastriate cortex, which is highly consistent with previous findings on vMMN to face (Kimura et al., 2012; Kovarski et al., 2021) or to other visual stimuli (e.g., Kimura et al., 2010; Urakawa et al., 2010; Susac et al., 2014). This suggests that MMN is modality specific (vMMN being elicited in visual areas while auditory MMN is elicited in auditory cortex— Näätänen et al., 2007) and relatively low-level (Susac et al., 2014).

Additionally, in the HSF condition, a more positive amplitude to deviant compared to equiprobable was observed in two time windows in a large cluster of electrodes over centro-parietal areas. This positive activity elicited by deviants was found in other studies (Knight, 1997; Stefanics et al., 2012; Csukly et al., 2013; Kovarski et al., 2017) and is thought to reflect the involuntary attention caught by the deviant stimulus, namely the P3a, with an activity elicited around 300–500 ms (Knight, 1997). Sources of this mismatch were found in a wide range of brain regions, from the right fusiform to the prefrontal and cingulate anterior regions, including the insula. Generators in temporal and limbic lobes were also described in other studies (Kimura et al., 2012; Li et al., 2012; Kovarski et al., 2021), as well as frontal activation (Kimura et al., 2010, 2012). The fusiform activity is in line with the preferential processing of faces (Kimura et al., 2012; Stefanics et al., 2012) especially in the right hemisphere, consistently with previous results on face vMMN (Kimura et al., 2012; Kovarski et al., 2021). Note that positive prefrontal activation is elicited in the second time window whereas occipito-temporal activation is elicited from the first steps of vMMN. Thus, Kimura et al. (2012) suggested that occipito-temporal changes might be related to prediction error signaling while later frontal activation might underline the update of predictive models. However, this hypothesis remains to be tested and discussed according to hierarchical predictive coding model (Friston, 2005, 2010). This model suggests hierarchical loops where prediction errors run bottom-up and update predictions at higher level, while top-down processes reduce predictions error at lower level (Friston, 2005; Garrido et al., 2009; Stefanics et al., 2014).

In the LSF condition, there was no significant time window where amplitude of the deviant was more negative than the stimulus presented in the equiprobable sequence. Nevertheless, the amplitude of the deviant was more positive than equiprobable over centro-parietal areas in a large time window. This result shows that LSF deviants, despite looking more similar to BSF than HSF deviants, are automatically detected as being different from BSF standards. However, the fact that LSF deviants did not elicit a significantly more negative occipital activity compared to equiprobable stimuli (contrary to HSF deviants), might indicate that they did not lead to similar prediction errors as HSF. In other words, the conflict between bottom-up sensory input and top-down predictions might be reduced in the LSF deviant condition. Again, this is in line with our hypotheses and with Bar's model, emphasizing the role of LSF information in visual processing by triggering predictions which are then used by a top-down process to facilitate recognition (Bar et al., 2006). Interestingly, source analysis for vMMN in LSF showed generators in the right anterior prefrontal cortex only, whereas the generators are widespread in the HSF condition, including temporal areas. This corroborates a different mismatch response to LSF vs. HSF, as already suggested by Susac et al. (2014), who interpreted this difference by the use of different streams for LSF and HSF, which is also in line with Bar's model.

Cluster analysis of the difference between dHSF-eHSF vs. dLSF-eLSF showed a significant difference only in the later stages. dHSF-eHSF elicited larger positive response than dLSF-eLSF over centro-parietal areas. This difference was due to the activity of the right fusiform, playing a crucial role in face processing. Additionally, dHSF-eHSF was more negative than dLSF-eLSF over left fronto-parietal areas, with a difference related to the activity of occipital and frontal areas. Again, it suggests that change detection in faces might be driven by HSF, but more specifically at the later stages of the processing, in line with the coarse-to-fine model of visual perception. Thus, while LSF would be needed in the early stages so that predictions facilitate face processing, HSF would be more involved later, when a detailed processing of faces is required.

### 4.2. Early Sensory Response

Visual inspection of topographies and ERPs of early sensory responses revealed a different activity for the P100 for HSF compared to LSF and BSF. The former exhibited a bilateral activation in parieto-occipital areas (no significant difference in amplitude according to SF was found over these areas) while the latter stimuli elicited responses over occipital sites as well, with a large positive peak. In the HSF condition, grand average ERP rather showed a large negativity at ≈130 ms. Interestingly, this large posterior negative response to HSF has been previously observed in several studies, usually peaking between 70 and 115 ms, especially in response to gratings (e.g., Kenemans et al., 2000; Ellemberg et al., 2002; Heslenfeld, 2003; Boeschoten et al., 2005) and to checkerboard stimuli (Kenemans et al., 2000). It has been suggested that the negative peak for HSF would reflect the parvocellular activity while the positive peak for LSF would reflect the magnocellular activity (Ellemberg et al., 2002). Larger P100 amplitude for LSF relative to HSF was found for faces in a gender categorization task (Jeantet et al., 2019), in a passive viewing task (Obayashi et al., 2009), and in a task of valence categorization during a rapid serial visual presentation (Tian et al., 2018), all including adults. Nevertheless, results are contradictory in the literature. Craddock et al. (2013) found larger P100 amplitude for HSF compared to LSF in a task involving gender categorization but with a different filtering (i.e., attenuation of some frequency bands) and a smaller sample. Pourtois et al. (2005), in a gender categorization task, showed reduced P100 amplitude to filtered stimuli of fearful and neutral faces compared to unfiltered but no significant difference between HSF and LSF. Variation in methodology (e.g., type of filtering, type of stimuli, contrast variation) might be responsible for such inconsistencies.

Moreover, the current study showed an advantage of HSF compared to LSF and BSF faces concerning the latency of the P100. This effect is surprising regarding our theoretical framework but the literature on this topic is heterogeneous. Indeed, it has been shown either shorter P100 latencies for LSF than HSF (Vlamings et al., 2009; Peters and Kemner, 2017) or no difference (Jeantet et al., 2019, for parieto-occipital channels) or, similarly to our results, shorter latencies for HSF than LSF (Obayashi et al., 2009; Jeantet et al., 2019, for occipital channels). However, similar or shorter latencies for the P100 in HSF compared to LSF does not rule out Bar's model. Indeed, the rapid extraction of LSF to generate prediction does not exclude a parallel extraction of HSF, suggested by other studies (Rotshtein et al., 2007; De Gardelle and Kouider, 2010). Different pattern of extraction could be related to the conscious or unconscious perception of the stimulus (De Gardelle and Kouider, 2010), but it would need further investigation. We can also hypothesize that after extraction, LSF might be rapidly processed by the dorsal pathway (on the basis of myelinated magnocellular layers) while HSF might be processed more slowly by the ventral pathway (see Nowak and Bullier, 1997; Chen et al., 2007). It should be noted that not only parietal regions are involved in the processing of LSF during face perception as processors has also been found in several other regions such as the middle occipital gyrus (Rotshtein et al., 2007), the fusiform face area, the occipital face area, the ventral lateral occipital complex (Goffaux et al., 2011) or also subcortical areas (Vuilleumier et al., 2003; McFadyen et al., 2017). The differences in topographies might also partly explain the differences in latencies between HSF and LSF for the P100. Studies which investigated the sources of P100 indicated that HSF and LSF ERPs might be generated in different areas of the visual cortex (Kenemans et al., 2000; Boeschoten et al., 2005). Their results show that LSF ERPs would have a neural orientation predominantly perpendicular to the scalp surface (presumably extra-striate), suggesting generators in the medial calcarine cortex or in V2. The orientation for HSF would be more parallel to the scalp surface (presumably striate source), suggesting generators in the middle occipital gyri. Theses effects appear to be robust across the two type of stimuli (gratings and checkerboard). Hence, different sources according to SF could explain differences in P100 topographies as well as differences in latencies because the use of different pathways for HSF and LSF (Skottun, 2015) can lead to different patterns of activation.

The N170, the specific ERP response to faces, was observed over the parieto-occipital areas in the three conditions. Results of statistical analyses showed no difference between conditions in latencies, contrary to other studies which show either faster latencies for LSF in a passive viewing task involving fearful and neutral faces (Peters and Kemner, 2017) or slower latencies for LSF in a gender categorization task (Jeantet et al., 2019). However, peak-to peak amplitudes were greater in HSF than in LSF and BSF. This latest result corroborates other recent findings on passive viewing (Obayashi et al., 2009; Mares et al., 2018), categorization (Jeantet et al., 2019) or detection tasks (Tian et al., 2018) and are in line with the coarse-to-fine integration for visual recognition and with Bar's model. In this framework, HSF would be preferentially used at later stages of visual recognition, for converging to a single percept. Thus, the strong activity elicited by HSF on the N170 could emphasize the analysis of face details required for a precise categorization of a face. Nevertheless, results also differ from previous studies which found no effect of spatial frequencies (Holmes et al., 2005) or larger amplitude for LSF compared to HSF during face processing (Goffaux et al., 2003; Pourtois et al., 2005; Halit et al., 2006). Inconsistencies across studies regarding differences in P100 amplitude or latencies according to SF might be related to differences in methodology either in the task and stimuli used or in the SF filtering choices and need to be understood by further investigations.

In sum, P100 appears more sensitive to LSF, while later and more face-specific processing, reflected by the N170 component, appears more sensitive to HSF. This pattern could be in line with the coarse-to-fine hypothesis of visual perception and more specifically with Bar's model. LSF would enable a global parsing of visual information at the early stages of visual processing, favoring predictions by a top-down process, whereas fine details conveyed by HSF would be integrated later, at a face-specific stage (Bar et al., 2006; Goffaux et al., 2011; Jeantet et al., 2019). This is in accordance with the vMMN results as HSF deviants elicited increased activity than HSF equiprobable in the fusiform area between 143 and 226 ms. Additionally, the difference between HSF and LSF mismatch response also showed the specific involvement of HSF at later stages (around 300–400 ms) again with an enhanced fusiform activity. Hence, vMMN to face appears to be related to the processing of HSF in face areas at advanced stages of visual perception. Bar et al. (2006) hypothesized that predictions would be made in the orbitofrontal cortex (OFC) as LSF elicited a higher signal than HSF in these regions, particularly around 115 ms. Their analyses showed synchrony between occipital areas and OFC beginning around 80 ms and between OFC and fusiform gyrus around 130 ms. Interestingly, P100 topographies in the present study also suggest a different activity in response to LSF vs. HSF in frontal areas as scalp topographies showed activation of these areas around 117 ms for LSF and BSF, but not for HSF. This could also support Bar's model, even if further investigation regarding sources and connectivity would be needed.

### 4.3. Limitations and Perspectives

The study has some limitations. First, the stimuli used were neutral faces stimuli. Other experiments are necessary to investigate if results could extend to emotional faces, but also to other type of complex stimuli (e.g., object, scenes).

Second, we based filtering cutoffs on those used in the literature, i.e., <1.5 cpd for LSF and >6 cpd for HSF. Nonetheless, according to the difference found between HSF and LSF, it could be of interest to explore vMMN variations with a different filtering (in terms of cutoffs and in terms of the type of filters used) to enhance our understanding of the results. Indeed, spatial filtering method employed in the current study presents several limits (Perfetto et al., 2020). Removing some low-level information leads to less ecological stimuli. Additionally, the use of Gaussian filters implies that HSF filtered images contain some amount of LSF (Perfetto et al., 2020), which can be a pitfall as the distinct pathways might not be clearly distinguished. Nevertheless, this did not seem to affect our results as sensory response analysis showed that LSF as BSF lead to a clear P100, while HSF lead to a N100. Thus, negative activity elicited by HSF faces might reflect the activity of the parvocellular pathway and our results are in line with a clear differentiation of the two pathways (Ellemberg et al., 2002). However, another limit is that HSF and LSF face stimuli presented important perceptual differences, with LSF being visually more similar to BSF than HSF, which could have led to more salient deviancy (as the filtering leads to darker background un the HSF condition, Perfetto et al., 2020). It is also worth noting that equalizing the contrast has the advantage to reduce differences in spectral energy between HSF and LSF, but results in non-natural amplitude spectra (Petras et al., 2019). Even if bias related to differences in characteristics of the stimuli raised in this section are limited here thanks to the controlled paradigm using an equiprobable sequence, other stimuli manipulations, such as the normalization procedure developed by Petras et al. (2019), or the use of reverted images of the complementary SF channel (Pourtois et al., 2005), could be used in future studies to further explore these issues. Additionally, it could help to investigate if differences between HSF and LSF in early sensory responses (P100 and N170) could be due to differences in spatial frequency spectral power. Nonetheless, we have to keep in mind that each method have advantages and weaknesses, and no method enables to control for perceptual factors associated with HSF and LSF stimuli while keeping the stimuli identical to natural ones.

Third, we used only one stimulus duration but further studies should investigate longer durations, in particular to compare vMMN to LSF vs. HSF vMMN. For instance, while 150 ms would be sufficient to process LSF and HSF information at a pre-attentive level, it might be insufficient for encoding HSF in perceptual representation (Gao and Bentin, 2011). As forming prediction implies memory processes, the impact of presentation time on the results should be further investigated. This could be performed by increasing the presentation time to 500 ms (Gao and Bentin, 2011), but the viability of such long presentation times in an MMN paradigm should be explored first.

Finally, O'Reilly and O'Reilly (2021) argued that equiprobable sequence would be an insufficient control because of long-term adaptation. The authors added that counterbalanced blocks would confound adaptation effects rather than eliminate them (O'Reilly and Conway, 2021; O'Reilly and O'Reilly, 2021). Accordingly, actual MMN paradigm, at least in the auditory domain, would not properly enable inferences about deviance detection and predictive coding because of state changes that can affect sensory responses. Even if our results are in line with previous findings and fit well with the predictive coding framework of MMN as well as with the predictive brain hypothesis, we cannot totally rule out that differences in sensory processing (see “Sensory Processing Theory” in O'Reilly and O'Reilly, 2021) or that another MMN framework such as the adaptation model (e.g., May, 2021) might partly explain our results. Indeed, despite intensively studied, MMN still poses a number of challenges in terms of interpretation (May, 2021).

## 5. Conclusion

This study is the first to investigate vMMN to spatially filtered faces and contributes to better understand how HSF and LSF are involved in automatic face processing. Our results suggest a predictive role of LSF, in line with the predictive coding framework of perception (Rao and Ballard, 1999; Friston, 2005, 2010; Bar et al., 2006), followed by HSF involved in face-specific processing.

## Data Availability Statement

The raw data supporting the conclusions of this article will be made available by the authors, without undue reservation.

## Ethics Statement

The studies involving human participants were reviewed and approved by Comite de Protection des Personnes Ile de France 1—IRB/IORG:IORG0009918, under agreement number 2019-A01145-52. The patients/participants provided their written informed consent to participate in this study.

## Author Contributions

AL, MG, MM, KK, DA, and FD contributed to the conception and design of the study. AL, SH, and LV collected the data and performed data analysis. AL, KK, SH, MG, and MM interpreted the data. AL, KK, SH, MG, MM, LV, and FD contributed to the manuscript. All authors contributed to the article and approved the submitted version.

## Funding

This study was supported by the French Ministry of Higher Education, Research and Innovation (France) to AL. This work has been partially supported by MIAI@Grenoble Alpes, (ANR-19-P3IA-0003) to MM. Data were acquired on a platform of France Life Imaging Network partly funded by the grant ANR-11-INBS-0006.

## Conflict of Interest

The authors declare that the research was conducted in the absence of any commercial or financial relationships that could be construed as a potential conflict of interest.

## Publisher's Note

All claims expressed in this article are solely those of the authors and do not necessarily represent those of their affiliated organizations, or those of the publisher, the editors and the reviewers. Any product that may be evaluated in this article, or claim that may be made by its manufacturer, is not guaranteed or endorsed by the publisher.
